# Comparative Outcomes of Doxorubicin and Cyclophosphamide with Sequential versus Concurrent Paclitaxel in the Adjuvant Treatment of Non-Metastatic Breast Cancer: A Cross-Sectional Analytical Study

**DOI:** 10.31729/jnma.9130

**Published:** 2025-06-30

**Authors:** Bishal Paudel, Bishnudutta Paudel, Rakshya Shrestha, Bishal Panthi, Ramila Shilpakar, Soniya Dulal, Sanjeev Kharel, Reechashree Dhungana, Bipsana Shrestha, Yogendra Prasad Singh

**Affiliations:** 1 Institute of Medicine, Tribhuvan University Teaching Hospital, Maharajgunj, Kathmandu, Nepal; 2 KIST Medical College and Teaching Hospital, Imadol, Lalitpur, Nepal; 3 National Academy of Medical Sciences, Mahabouddha, Kathmandu, Nepal; 4 B. P. Koirala Institute of Health Science, Dharan, Nepal; 5 Health Directorate, Ministry of Health, Bagmati Province, Hetauda, Nepal.

**Keywords:** *breast cancer*, *chemotherapy*, *cyclophosphamide*, *doxorubicin*, *paclitaxel*

## Abstract

**Introduction:**

Studies have compared the efficacy and toxicities of doxorubicin and cyclophosphamide every three weeks for four cycles followed by four cycles of paclitaxel every three weeks (AC/T); with paclitaxel, doxorubicin, and cyclophosphamide (TAC) every three weeks for six cycles for adjuvant treatment of breast cancer in western countries. Genetic and environmental disparities in Nepalese population warrant the need for similar studies in Nepal. This study compares the toxicity patterns and compliance of AC/T versus TAC in the adjuvant treatment of non-metastatic breast cancer in Nepalese women.

**Methods:**

A hospital-based cross-sectional analytic study was conducted at Bir Hospital, Kathmandu after obtaining the ethical approval (Reference number: 931/076/077). Sixty women who completed either AC/T or TAC regimens were evaluated. Confounding was minimized by strict inclusion/ exclusion criteria (restriction), group matching, and random sampling. Primary outcome was grade 3-4 hematological toxicity; secondary outcomes included other adverse effects and compliance.

**Results:**

Although hematological toxicities were higher in the TAC group, differences were not statistically significant. Non-hematological toxicities (fatigue, nausea, vomiting, pain, nail changes) were significantly higher in the TAC group. Edema was more prevalent in the AC/T group (p=0.04). Compliance without modification favored AC/T (64.5% vs. 34.5%; p=0.038).

**Conclusions:**

Sequential AC/T demonstrated superior tolerability and compliance. Confounder control through study design and statistical methods strengthens the validity of these findings, though larger studies are warranted.

## INTRODUCTION

Breast cancer poses significant health challenges worldwide, including in Nepal.^1,2^ Global Cancer Observatory (GLOBOCAN) 2022 reports breast cancer as the most common cancer in Nepalese women, with 2255 new cases and 1149 deaths annually.^2^ Adjuvant chemotherapy with polychemotherapy reduces recurrence and improves overall survival. Taxane and anthracycline-based regimens have shown better outcomes.^3,4^ However, chemotherapy causes adverse effects, often underestimated outside clinical trials.^5^

The BCIRG-005 trial compared doxorubicin and cyclophosphamide followed by paclitaxel (AC/T, sequential) with paclitaxel, doxorubicin, and cyclophosphamide (TAC, concurrent) for nodepositive breast cancer.^6^ Its 10-year analysis confirmed comparable efficacy.^7^ Genetic, environmental, lifestyle, and socioeconomic factors may influence chemotherapy outcomes, necessitating similar studies in Nepalese women.^6,7,8^

Given this context, the present study compares the toxicity profile and compliance of AC/T versus TAC in non-metastatic breast cancer, focusing on hematological toxicities, infections, non-hematological toxicities, and treatment compliance.

## METHODS

A hospital-based cross-sectional analytic study was conducted in the Clinical Oncology Unit at Bir Hospital, National Academy of Medical Sciences (NAMS), Kathmandu, Nepal. Data collection was carried out from November 1, 2019, to May 1, 2020. Ethical approval was obtained from the Institutional Review Board of NAMS (Reference number: 931/076/077). Written informed consent was obtained from all participants. Patient identity and personal information were deidentified by assigning a unique identification number to each participant. The study was conducted in accordance with the Declaration of Helsinki and the principles of good clinical practice.

The sample size was calculated to detect a 25% difference in grade 34 hematologic toxicities between the TAC and AC/T groups. Based on early reports of high hematologic toxicity with taxane-containing regimens, particularly in settings without routine prophylactic growth factors, we assumed an incidence of approximately 50% in the TAC group and 25% in the AC/T group.^3,4^ The level of significance was set at 5% (two-sided), and the study was powered at 80%. Based on these assumptions, a total of 55 participants (2728 per group) were required. To account for potential dropouts, 60 women were enrolled, with 29 in the TAC group and 31 in the AC/T group.

Confounding was minimized through the use of strict inclusion and exclusion criteria: women aged 18-70 years with histologically confirmed unilateral operable breast carcinoma (T1-3, N0-1, M0), ECOG performance status 0-2, and no significant comorbidities. Patients with T4 or metastatic (M1) disease, renal, hepatic, or cardiac dysfunction, or uncontrolled serious medical conditions were excluded. Groups were balanced on age, menopausal status, smoking history, and disease characteristics. Eligible patients were selected through simple random sampling to reduce selection bias.

Data were collected using a structured proforma that included demographic details, clinical variables, chemotherapy regimen, adverse effects (as per CTCAE v4.0), and treatment compliance. Data analysis was performed using SPSS version 22. Descriptive statistics were used to summarize patient characteristics. Fisher's exact test was used to compare proportions. A p-value of <0.05 was considered statistically significant.

## RESULTS

A total of 60 women participated in the study, with 31 women in AC/T and 29 in TAC arms. The baseline characteristics were comparable in both groups (Table 1). Notably, 37 of these 60 women (61.7%) were illiterate.

**Table 1:** Characteristics of breast cancer patients (N=60).

Age Group (in years)	AC/T (n=31) n(%)	TAC(n=29) n(%)
<30	0	3(10.34%)
30-39	5(16.13%)	5(17.24%)
40-49	10(32.26%)	10(34.48%)
50-60	10(32.26%)	7(24.14%)
>60	6(19.35%)	4(13.79%)
Smokers	14 (45.1%)	10 (34.5%)
Post-menopausal	16 (51.6%)	14(48.3%)

AC/T-doxorubicin and cyclophosphamide followed by paclitaxel (Sequential; TAC-paclitaxel, doxorubicin, and cyclophosphamide (TAC, concurrent)

The rates of red cell transfusion, febrile neutropenia and asymptomatic neutropenia were all higher for concurrent paclitaxel (i.e TAC arm), though not statistically significant. (Table 2) The secondary end points studied were rates of documented infection, compliance factors and non-hematological toxicities. (Table 2). The rate of documented infection was only slightly higher in the TAC arm and statistically insignificant. Statistically significant association for compliance was seen when it came to completion of all cycles without modification favoring the AC/T regimen (p=0.038) which also weighed higher in terms of number of women completing all cycles, the latter statistically insignificant.

Among the non-hematological toxicities, TAC regimen demonstrated superiority only in terms of significantly lower rates of edema (p=0.04). The rates of fatigue, nausea, vomiting, nail changes and pain showed significantly higher occurrence in the TAC arm. Other adverse effects like diarrhea, dyspepsia, anorexia and depressed ejection fraction were also reportedly higher in the TAC arm, though statistically insignificant.

**Table 2:** Comparison of primary and secondary endpoints between AC/T and TAC (N=60)

Outcome	AC/T (n=31)	TAC (n=31)	Total (N=60)	p value
**Hematological Toxicities**				
Red cell Transfusion	6 (19.4%)	8 (27.6%)	14 (23.3%)	0.547
Febrile Neutropenia	4 (12.9%)	6 (20.7%)	10 (16.7%)	0.500
Asymptomatic Neutropenia	11 (35.5%)	17 (58.6%)	28 (46.7%)	0.120
Infection rates (documented in blood culture)	3 (9.7%)	4 (13.8%)	7 (11.7%)	0.702
**Compliance factors**				
Completion of all cycles of proposed regimen	29 (93 5%)	22 (75.9%)	51 (85%)	0.076
Completion of all cycles without modification	20 (64.5%)	10 (34.5%)	30 (50%)	0.038
**Non-hematological toxicities**				
Fatigue	19 (61.3%)	29 (100.0%)	48 (80.0%)	<0.001
Nausea	19 (61.3%)	28 (96.5%)	47 (78.3%)	0.001
Vomiting	11 (35.5%)	22 (75.9%)	33 (55.0%)	0.002
Diarrhea	14 (45.2%)	18 (62.1%)	32 (53.3%)	0.208
Dyspepsia	13 (41.9%)	19 (65.5%)	32 (53.3%)	0.077
Anorexia	27 (87.1%)	28 (96.5%)	55 (91.7%)	0.355
Nail Changes	8 (25.8%)	16 (55.2%)	24 (40.0%)	0.034
Edema	17 (54.8%)	8 (27.6%)	25 (41.7%)	0.040
Pain	3 (9.7%)	16 (55.2%)	19 (31.7%)	<0.001
Depressed cardiac ejection fraction (up to 45%)	0	2 (6.9%)	2 (3.3%)	0.229

AC/T-doxorubicin and cyclophosphamide followed by paclitaxel (Sequential; TAC-paclitaxel, doxorubicin, and cyclophosphamide (TAC, concurrent)

## DISCUSSION

Breast cancer, the second most common cancer worldwide, poses an enormous burden in lower middle-income countries (LMICs).^1^ Presentation at an advanced age is an added obstacle to breast cancer care in LMICs.^9^ The astounding majority of illiterate patients (61%) in this study points towards the probable reason for late presentation in Nepal. Many other studies done in countries like Iran and Netherlands support this finding. They suggest that the knowledge and education acts as a determining factor for presenting late with their symptoms in the hospital in cancer patients. This delay has been associated with the cognitive interpretation of the cancer symptoms by patients which is less likely in patients with lower education. For an instance, patients who were suspected of having cancer and who had knowledge about that cancer, were more likely to follow up with their doctors if their symptoms did not resolve in time. However, the studies did not state about the time duration the patient waited between first time noticing their symptoms and visiting their doctors for consultation.^10^ Further, stress and fear was also mentioned as one of the factors leading to delay in consulting doctors in studies done in different parts of world including England. Stress and fear were mainly due to past experience of family member who were diagnosed with cancer and had a painful death, or due to the fear of getting diagnosed with cancer.^11,12^ Moreover, one of the studies found that consultation delay was found to be seven times higher in patients who reported stress during the time period prior to their diagnosis.^13^

The Breast Cancer International Research Group (BCIRG-005) trial compared the efficacy and toxicity of doxorubicin and cyclophosphamide every three weeks for four cycles followed by four cycles of paclitaxel every three weeks (AC/T i.e sequential paclitaxel); with paclitaxel, doxorubicin, and cyclophosphamide (TAC i.e. concurrent paclitaxel) every three weeks for six cycles for adjuvant treatment of early node positive breast cancer.^14^ The ten year analysis of the BCRIG 005 trial comparing the long term outcomes confirmed comparable efficacy of the two regimen.^15^ However, both the regimen showed different toxicity profiles in the treatment of early node-positive breast cancer with TAC showing higher rates of febrile neutropenia and thrombocytopenia and AC/T showing greater nail changes, myalgia, sensory neuropathy and fluid retention.^14,15^ This study was similar in reporting higher edema rates in AC/T and higher neutropenia in TAC, though the latter was not statistically significant.

The NSABP B38 study had also reported comparable efficacy across these two regimens.^16^ In NSABP B38, TAC showed greater febrile neutropenia and diarrhea while AC/T showed significantly higher anemia (Hb <8g/dl) and erythropoietin use, myalgias and sensory neuropathies.^16^ In our study, red cell transfusion and neutropenia rates was both higher in the TAC arm, though statistically insignificant.

Neutropenia, a worrisome hematological adverse effect in breast cancer patients, precipitated by myelosuppression has been often reported 10-14 days after each cycle of adjuvant chemotherapy.^17^ Chemotherapy induced neutropenia (CIN) is considered the most common toxicity as a result of anticancer drugs administration. A large prospective registry shows that 37% of breast cancer patients experience absolute neutrophil count (ANC) less than 500 cells per cubic mm over first four cycles of chemotherapy. Approximately 70% of these initial episodes of neutropenia occur during first cycle.^18,19^

However, in most women, the white blood cell count raise before the next cycle of chemotherapy.^17^ The most important patient-related factor for developing risk of CIN is increased age. Hence, elderly patients diagnosed with breast cancer and undergoing chemotherapy have been observed to be more prone to neutropenia than the younger patients.^20^ Neutropenia following chemotherapy was seen in 46% of our study participants. The combination of docetaxel and doxorubicin has been linked to deaths due to serious sepsis in patients with febrile neutropenia.^21^ A total of seven (11.7%) patients demonstrated infection in blood culture in our study, with no group significantly predisposed. Another study investigating blood cultures in patients receiving chemotherapy suggested that positive blood culture is not that common adverse effects of treatment (only 3% incidence rate in their sample population), however, if they occurred, higher mortality rate (24% in their study) has to be noted. Moreover, the higher incidence of positive blood culture was observed in the first three months of starting the cancer treatment. It might be due to the reason that patients at this time period are more likely to be severely immunosuppressed due to treatment. Additionally, close monitoring during that time period and more contact with health professionals might also explain higher incidence of Positive blood culture.^22^

The most common adverse effects reported in this study was anorexia, fatigue, nausea and vomiting which is comparable to findings from larger trials.^14,15,16^ The combination of doxorubicin and cyclophosphamide has been previously established as highly emetogenic.^23^ Doxorubicin in particular is also considered cardiotoxic.^17^ This could account for depressed cardiac ejection fraction (up to 45%) seen in two patients in the TAC group.

The study assessed compliance in two domains: completion of all chemotherapy cycles and completion without modification. AC/T regimen had higher completion rates in both domains, with 93.5% versus 75.9% for all cycles and 64.5% versus 34.5% for completion without modification, showing statistical significance in the latter.

Modification included dose and schedule adjustments to promote adherence in the face of intolerable effects.

This study favored sequential paclitaxel as opposed to concurrent administration, the former less toxic and more conducive to compliance. Despite the need for multiple hospital visits for chemotherapy administration, patients adhered more to sequential paclitaxel regimen. The lesser rate of myelosuppression could also render it more cost effective, though a larger sample size is necessary to explore potential cost-effectiveness. The smaller sample size cautions against generalizability of the study findings, which remains the major limitation of this study. Larger scale studies are warranted to analyze cost effectiveness and draw definite conclusions for safety and efficacy profiles to better inform future directions for breast cancer chemotherapy in Nepalese women.

## CONCLUSIONS

The toxicity profiles of sequential and concurrent paclitaxel regimens were consistent with other studies. The AC/T regimen is more favorable in terms of better compliance and lesser adverse effects compared to the TAC regimen.
